# Minute Cellular Nodules as Early Lesions in Rats with Silica Exposure via Inhalation

**DOI:** 10.3390/vetsci9060251

**Published:** 2022-05-25

**Authors:** Yaqian Li, Fuyu Jin, Tian Li, Xinyu Yang, Wenchen Cai, Shifeng Li, Xuemin Gao, Na Mao, Heliang Liu, Hong Xu, Fang Yang

**Affiliations:** Hebei Key Laboratory for Organ Fibrosis Research, School of Public Health, North China University of Science and Technology, Tangshan 063210, China; lyqewbar@163.com (Y.L.); fuyujinjfy@163.com (F.J.); tiantian__1997@163.com (T.L.); zwns69618@163.com (X.Y.); chenwencai1991@163.com (W.C.); leimengpi@163.com (S.L.); gaoxm0623@163.com (X.G.); namao1991@163.com (N.M.); 13933499300@163.com (H.L.)

**Keywords:** rats, silicosis, macrophage alveolitis, cellular nodules, fibrotic cellular nodules

## Abstract

Mechanisms of silicosis have yet to be clarified, and pathological conditions are inaccurately described in some experimental studies on silicosis. This study was aimed at describing initial lesions in silicosis, as observed in rats with silica exposure via inhalation, and major histopathologic alterations. Male Wistar rats were exposed to silica for 24 weeks. Hematoxylin and eosin staining indicated the presence of “cellular nodule+ macrophage alveolitis” in rats exposed to silica from the 2–16 weeks time points and “fibrotic cellular + cellular nodule” in rats exposed to silica via inhalation for 24 weeks. By immunohistochemistry, the following were noted: a continual increase in the positive expression of CD68 in macrophages in the lungs of rats exposed to silica; hyperplasia in alveolar type II cells (AT2); loss of original phenotypes in fibrotic cellular nodules, macrophages, and AT2 cells; loss of endothelial cells in silicotic nodules; and positive expression of α-smooth muscle actin in macrophages. Typical pathological changes in silicosis were also summarized. Among these changes were macrophage alveolitis, cellular nodules, and fibrotic cellular nodules, including an increase in minute cellular nodules in the early stages and the formation of fibrotic cellular nodules in the late stages.

## 1. Introduction

Silicosis is caused by the inhalation of respirable crystalline silica dust, which leads to progressive, irreversible, and fatal inflammation and fibrosis of the lung over time [1]. No specific treatment for this condition has thus far been identified, although a small number of patients may be offered a lung transplant [2].

Multiple omics approaches, including genomics, epigenomics, transcriptomics, proteomics, and metabolomics, have bridged underlying molecular alterations with the initiation and progression of silicosis [3,4,5,6]. Most of these studies have employed bolus exposure of mice to silica, followed by the evaluation of various pulmonary parameters, including lung function, bronchoalveolar lavage fluid test, serum test, collagen deposition, myofibroblast differentiation, e.g., at selected times post-exposure. Antifibrotic drugs, including pirfenidone and nintedanib, which are approved by the United States Food and Drug Administration for idiopathic pulmonary fibrosis, have been widely reported to alleviate silicotic models [7,8]. Tetrandrine as a treatment for silicosis has been approved in China [9] and can inhibit canonical and noncanonical NLRP3 inflammasome activation in lung macrophages [10]. However, all previous studies on therapeutic targets for silicosis have been conducted after the formation of large silicon nodules. Drug intervention after the formation of fibrosis nodules can only alleviate disease progression and cannot reverse the formation of silicon nodules and collagen deposition. No large, randomized, and placebo-controlled clinical trials have thus far been conducted to assess the effect of these drugs on silicosis, and the safety and reliability have yet to be determined. The major problem to address is that the mechanisms of silicosis remain unclear. Specifically, pathological conditions are inaccurately described in some experimental studies on silicosis.

A recent correspondence article has proposed that most pathogenic processes in silicosis are largely based on putative mouse “models” of human disease and are not effective for use in humans. Further, in silicosis, the classic pathology needs to be inducible only via the inhalation of freshly cleaved quartz particles [11]. An inhalation-induced silicosis rat model can well simulate the progression of human silicosis. In a few published literature reviews, only early black and white images are presented, and a description of typical nodule morphology is lacking. By using typical markers, the present study described an initial lesion in silicosis, as observed in rats exposed to silica via inhalation, and some major histopathologic alterations.

## 2. Materials and Methods

### 2.1. Animal Experiments

A silicotic rat model was established using a HOPE MED 8050 exposure control apparatus (HOPE Industry and Trade Co. Ltd., Tianjin, China) reported in previous research [12,13,14]. All animal experiments were approved by the North China University of Science and Technology Institutional Animal Care and Use Committees (2013-038 and LX2019033) and complied with the United States National Institutes of Health Guide for the Care and Use of Laboratory Animals.

The silica used In this study was silicon dioxide (SiO_2_, s5631, Sigma-Aldrich, St. Louis, MD, USA). The silica was fractured in an agate jar for 2 h and rendered endotoxin-free by baking at 180 °C for 6 h. Male Wistar rats (3 w-of-age) were from Vital River Laboratory Animal Technology Co. Ltd. (SCXY 2016-0008, Beijing, China). Upon arrival, all rats were housed in a specific pathogen-free facility. They were maintained in a 12 h/12 h light/dark cycle and provided with food and water ad libitum. The rats were randomly divided into two groups (n = 10 each): Rats in a HOPE MED 8050 inhalation chamber measuring 3 m^3^ were exposed to filtered air (the control group), whereas those kept in another chamber were exposed to 50 ± 10 μg/m^3^ silica for 3 h/d for 2, 4, 8, 12, 16, and 24 weeks. Chamber atmospheres were maintained under the following conditions: temperature, 20–25 °C; humidity, 70–75%; pressure −50 to +50 Pa; oxygen concentration 20%; and SiO_2_ mixture flow rate 3.0–3.5 mL/min. SiO_2_ concentration in the device was detected every 2 weeks to maintain the SiO_2_ concentration. The rats were sacrificed after 24 weeks of treatment. Their lungs were isolated, and their lung tissues were fixed with a 4% paraformaldehyde solution. By being soaked in progressively lower concentrations of ethanol, the samples were sequentially dehydrated, embedded in paraffin, and cut into sections with a thickness of 5 μm for staining. 

### 2.2. Histological Examination of Lung Tissue

The sections were deparaffinized and stained with hematoxylin and eosin (HE staining; BA4025, BaSO Diagnostics Inc., Zhuhai, China), periodic acid–Schiff (PAS) (G1281, Solarbio, Beijing, China) staining was used to detect lipoprotein deposition, and observed under a light microscope. Collagen accumulation was assessed by morphometric analysis of Van Gieson staining (VG staining; BA4084, BaSO Diagnostics Inc., Zhuhai, China) according to the manufacturer’s instructions.

### 2.3. Immunohistochemistry and Immunofluorescence Staining

Immunohistochemistry (IHC) was performed using published protocols [13]. Antigens were retrieved under high-temperature and high-pressure conditions and then blocked with H_2_O_2_ to quench the endogenous peroxidases. Samples were incubated with antibodies directed against ATP-binding cassette subfamily A member 3 (ABCA3, 1:200 dilution, ab24751, Cambridge, UK) [15], α-smooth muscle actin (α-SMA, 1:200 dilution, ab5694 and ab7817, Cambridge, UK), CD34 (1:200 dilution, ab81289, Cambridge, UK), CD68 (1:200 dilution, ab201340, Cambridge, UK), and Na^+^-K^+^-ATP (1:200 dilution, ET1069-76, HUABIO, Hangzhou, China) at 4 °C overnight. Subsequently, the samples were incubated with a secondary antibody (PV-6000, Beijing Zhongshan Jinqiao Biotechnology Co. Ltd.,China) at 37 °C for 30 min. Immunoreactivity was visualized with 3,3′-diaminobenzidine (DAB; ZLI-9018, ZSGB-BIO, Beijing, China), and brown staining was considered positive. In immunofluorescence staining, the samples were incubated with antibodies directed against vimentin (1:500 dilution, ab92547, Cambridge, UK) and ABCA3 (1:200 dilution) at 4 °C overnight. They were then conjugated with goat anti-rabbit IgG (H + L) TRITC (130,154, SeraCare, Milford, MA, USA) and goat anti-mouse IgG (H + L) FITC (121,051, SeraCare, Milford, MA, USA) at 37 °C for 1 h. The cell nuclei were stained with a 1:500 dilution of 5 mg/mL 4′,6-diamidino-2′-phenylindole (DAPI; 14,285, Cayman Chemical Company, Ann Arbor, MI, USA) for 5 min. The colocalization of vimentin and ABCA3 was observed under an Olympus DP80 microscope (Olympus, Hamburg, Germany), and the images were analyzed with the cellSens Imaging Software version 1.8 (Olympus, Hamburg, Germany). Red fluorescence indicated ABCA3 expression, and green fluorescence indicated vimentin expression; silica in cells was observed under a polarized light microscope (Olympus, Hamburg, Germany). Image Pro plus 6.0 was used to delineate and calculate the number and area of silicon nodules.

## 3. Results

### 3.1. Chronic Inhalation of Silica Induced Progressive Pulmonary Fibrosis

Numerous studies have reported that inhaled silica can induce biphasic pulmonary responses in rats, indicating “inflammation” and “fibrosis” stages in rats exposed to silica [16,17]. The major histopathologic alterations are alveolitis, alveolar type II cells (AT2) hyperplasia, lipoproteinosis, and interstitial fibrosis [18].

In the present study, the gross appearance of a representative lung in Week 2 was smooth and relatively normal, or some small silicon nodules were present. An increasing number of silicon nodules were noticeably scattered in the lung in Week 12. In Week 24, silicon nodules disappeared, and the texture of the lungs toughened; similar “biphasic pulmonary responses” in rats after silica inhalation were also observed, including “cellular nodule + macrophage alveolitis” in rats exposed to silica for 2–16 weeks and “fibrotic cellular + cellular nodules” in rats that inhaled silica for 24 weeks. Most substantial histopathologic alterations could be observed in the rats that inhaled silica in Week 24 (Supplementary Figure S1).

**Figure 1:**
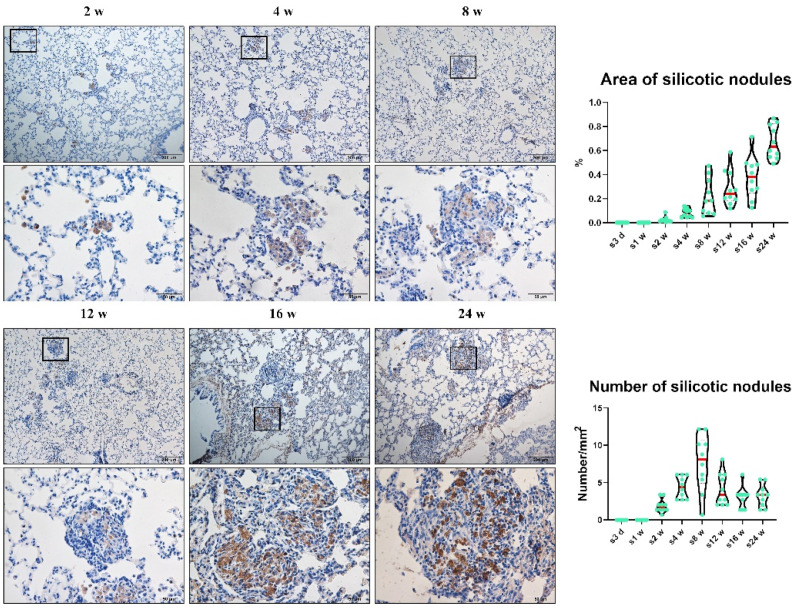
Positive expression of CD68 in macrophages in rats exposed to silica for 2, 4, 8, 12, 16, and 24 weeks. As the silica exposure time is extended, the positive expression of CD68 in macrophages continually increases. n = 10 per group, (Bar = 500 μm, 50 μm).

CD68 was also used to mark macrophages by IHC. A continual increase in positive CD68 expression was also found in macrophages in the lungs of rats exposed to silica (Figure 1). The earliest cellular nodules were found in rats exposed to silica via inhalation for 2 weeks and involved one alveoli. In the present study, these cellular nodules consisted of several closely adjacent macrophages, and macrophage alveolitis consisted of multiple free macrophages in the alveolus or septum (Supplementary Figure S2).

The number of silicotic nodules increased and reached its peak at Week 8, and the area of the silicotic nodules was enlarged in rats as the time of silica inhalation increased (Figure 1). These initial cellular nodules and their surrounding alveolus are basic lesions in silicotic rats exposed to silica via inhalation at the 2–24 weeks time points. Pulmonary alveolar proteinosis is associated with defects of alveolar macrophage phagocytosis and bactericidal activities. By phagocytosing microbes, dead cells, and other airborne particles, macrophages maintain lung homeostasis to prevent unnecessary inflammation. A deficiency in granulocyte-macrophage colony-stimulating factor signaling can lead to the dysregulation of macrophage surfactant clearance and causes the accumulation of proteins and phospholipids in air spaces, leading to pulmonary alveolar proteinosis [19]. Collagen deposition measured by VG staining and lipoproteinosis determined by PAS staining were conducted on rats exposed to silica in Week 24. With the extension of silica exposure time, collagen deposition and lipoproteinosis became increasingly prominent. Lung biopsy showed the nearly complete filling of alveoli with acellular, eosinophilic, and PAS-positive sediment (Supplementary Figure S3).

### 3.2. Hypertrophy and Hyperplasia of AT2 Cells

**Figure 2:**
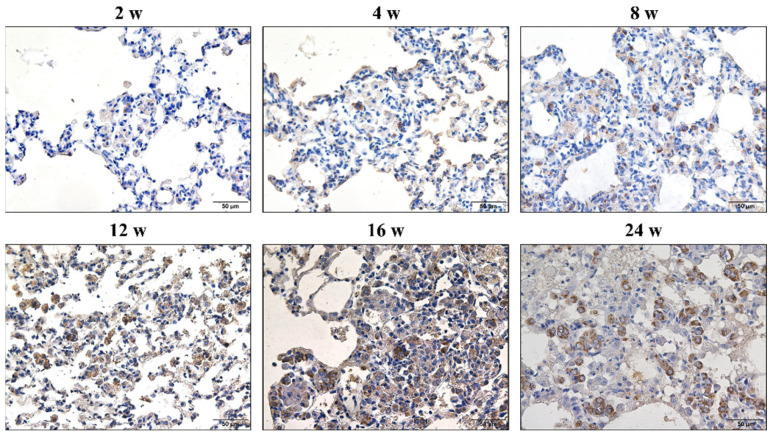
Positive expression of ATP-binding cassette subfamily A member 3 (ABCA3) in alveolar type II cells (AT2 cells) in rats exposed to silica for 2, 4, 8, 12, 16, and 24 weeks. Initial cellular nodules appear at 2 weeks. AT2 cells show hypertrophy and hyperplasia. At the 2–16 weeks time points, hypertrophy and hyperplasia in the AT2 cells are restricted to cellular nodules, and cellular nodules with macrophages are formed. (Bar = 50 μm).

These initial cellular nodules, which appeared at the earliest time point (Week 2), were consistently associated with hypertrophy and hyperplasia of the AT2 cells (Figure 2). At the 2–16 weeks time points, hypertrophy and hyperplasia of the AT2 cells were restricted to cellular nodules, and cellular nodules with macrophages were formed. Previous studies have indicated the involvement of epithelial–mesenchymal transition (EMT) in the development of progressive massive pulmonary and hepatic fibrosis [20,21,22,23]. These initial minute cellular nodules, which appeared at the earliest time point (Week 2), were consistently associated with hypertrophy and hyperplasia of the AT2 cells (Figure 2). However, in the present study, it is hard to find a typical EMT process (Supplementary Figure S4). S100A4 (also known as fibroblast-specific protein 1) was used as a fibroblasts marker in a previous study [20] to indicate fibroblast–myofibroblast differentiation and was found to exhibit positive expression in macrophages in rats exposed to silica from 2–24 weeks (Supplementary Figure S5).

### 3.3. α-SMA Was Positively Expressed in Macrophages and Smooth Muscle Cells

**Figure 3:**
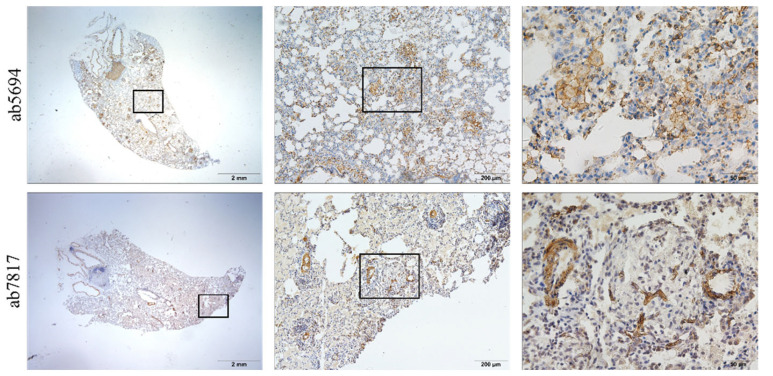
Positive expression of α-smooth muscle actin (α-SMA) in silicotic nodules in rats exposed to silica for 24 weeks. In the same sections, α-SMA is positively expressed in macrophages and located in the membrane similar to “actin rings” (above); by using another antibody, positive expression of α-SMA is exhibited only in smooth muscle cells in blood vessels and trachea (below). (Bar = 2 mm, 200 μm, and 50 μm).

The positive expression of α-SMA has been observed in the primary culture of rat lung fibroblasts induced by transforming growth factor-β and in silicotic nodules [24]. Contrary to a previous report [25], the silicotic lesions were rich in the macrophages with α-SMA positive expression but not in AT2 cells. Positive expression of α-SMA was also found in macrophages and located in the membrane similar to “actin rings” stained by phalloidin, another commonly used marker for myofibroblasts. Notably, the positive expression of α-SMA was only observed using another antibody in smooth muscle cells (Figure 3). However, these antibodies were positively expressed in lung fibroblasts in vitro [12,24].

**Figure 4:**
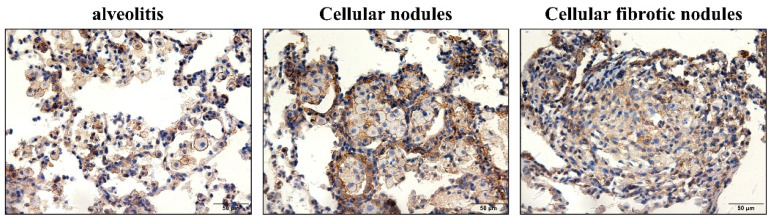
Positive expression of N+-K+-ATP in rats exposed to silica for 24 weeks. In alveolitis and cellular nodules, the membrane structure of all cells remains intact, and the cell membrane of macrophages and AT2 cells are lost in fibrotic cellular nodules. (Bar = 50 μm).

**Figure 5:**
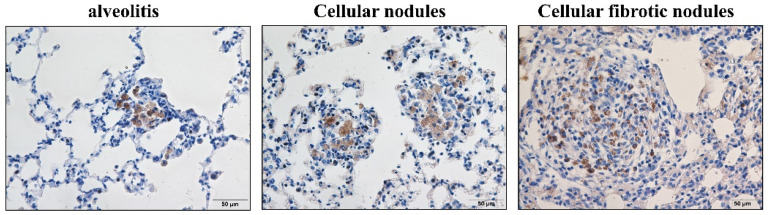
Positive expression of CD68 in silicotic rats exposed to silica. Macrophages are concentrated in nodules. In alveolitis cellular nodules and cellular nodules, the proportion of CD68-positive cells in nodules is decreasing. (Bar = 50 μm).

**Figure 6:**
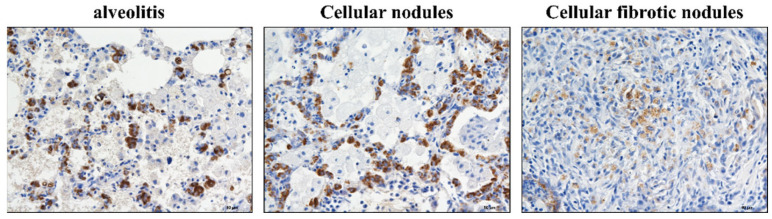
Positive expression of ABCA3 in silicotic rats. In alveolitis and cellular nodules, AT2 cells show hypertrophy and hyperplasia in fibrotic cellular nodules, and the positive expression of ABCA3 in AT2 cells is decreased. (Bar = 50 μm).

**Figure 7:**
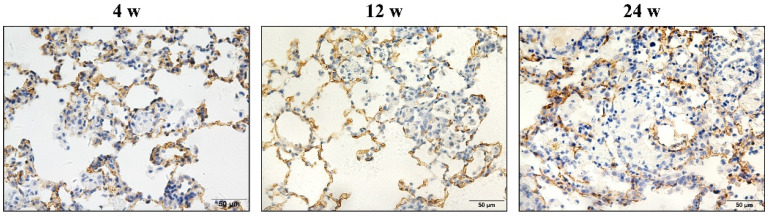
Positive expression of CD34 in endothelial cells in rats exposed to silica for 4, 12, and 24 weeks. With the extension of silica exposure time, the proportion of CD34 positive cells in nodules decreases, and pulmonary vascular endothelial cells are lost in silicotic nodules. (Bar = 50 μm).

**Figure 8:**
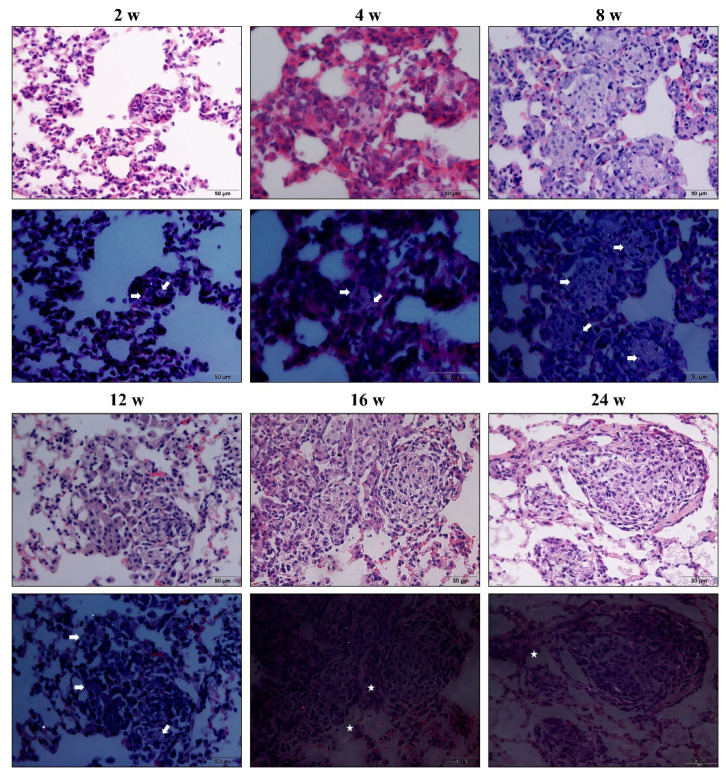
HE staining (above) and images under the polarized light lens (below) of rats exposed to silica for 2, 4, 8, 12, 16, and 24 weeks. With the extension of silica exposure time, the silicon nodules became larger; under the polarized light lens, macrophages in the nodules contain silica from Week 2 to Week 12 (arrow); meanwhile, macrophages around nodules not containing silica are observed from Week 16 to Week 24 (star). (Bar = 50 μm).

Both macrophages and AT2 cells lost their original phenotype in fibrotic cellular nodules. Macrophages lost not only their cell membrane (Figure 4) but also lost the positive expression of CD68, particularly in cellular fibrotic nodules (Figure 5); the same was true for the ABCA3 of AT2 cells. (Figure 6). Endothelial-to-mesenchymal transition has also been reported to occur during bleomycin-induced pulmonary fibrosis [26], indicating that endothelial cells are also a source of myofibroblasts. In the present study, CD34 was used to mark pulmonary vascular endothelial cells and was lost in silicotic nodules, indicating the presence of ischemic hypoxia in the formation of silicotic nodules (Figure 7). Notably, when we used a polarization microscope to observe the lung sections, all macrophages observed in the early stages of the lesion contained silica. As the disease progressed, we observed macrophage alveolitis around the formed fibrous nodules in the lung sections of Week 24 rats, and some of the cells did not contain silica (Figure 8).

## 4. Discussion

Several studies have well documented that silica inhalation promotes pulmonary inflammation and fibrosis in rats and that recovery after silica exposure promotes lung fibrosis. The fundamental concept to explain the pathogenesis of silicosis is relatively simple: a chronic inflammatory status (referred to alveolitis), in which the immune cells are active and release toxic mediators, damages the pulmonary architecture and modulates the accumulation of mesenchymal cells and their connective tissue product. Initially, pulmonary defense mechanisms were able to compensate for and control silica-induced pulmonary damage until a rather defined point in the exposure. Despite the absence of particle overload, the progressive development of alveolar hypertrophy and hyperplasia, lipoproteinosis, and pulmonary fibrosis occurred. Alveolar lipoproteinosis is a well-documented response to inhaled silica [16,17,18,27]. These studies provide accurate figures describing pathological changes in silicosis but lack images of silicosis progression. By using HE and VG staining, silicotic nodules have been assigned various grades—such as cellular nodules (stage I), fibrotic cellular nodules (stage II), cellular fibrotic nodules (stage III), and fibrotic nodules (stage IV) [28]—but they are still difficult to distinguish. Other studies have applied unsupervised clustering analysis to define the pathophysiological process of silicosis as that which consists of four stages: the normal, inflammatory, progressive, and fibrotic stages [6]. However, reports suggest that typical silicotic nodules are formed during the inflammatory stage. Some studies also provide blurry images, which fail to distinctly show the pathological changes that occur in silicosis [29,30]. In 2015, some pathologists summarized the principle of silicon nodule enlargement as follows: macules—small airway lesions (bronchiolitis caused by mineral dust exposures)—silicotic nodule—progressive fibrosis [31]. In the present study, the early lesions of silicosis occurred in one alveoli, referred to as a minute cellular nodule consisting of several macrophages containing silica.

Macrophages and alveolar epithelial cells are the first cell types in contact with silica. They are critical effector cells and the first defense against foreign substances in pneumoconiosis. Cross-talk between alveolar and lung epithelial cells is essential to maintaining lung homeostasis [32]. Single-cell RNA-Seq data suggested that secreted phosphoprotein 1 (SPP1) expression was increased specifically in alveolar macrophages during fibrosis; meanwhile, chitinase-3-like protein 1 (CHI3L1) was increased in both alveolar macrophages and AT2 cells. Moreover, a previous study confirmed the predicted emergence of CHI3L1- and SPP1- positive alveolar macrophages and increased CHI3L1 expression in AT2 cells in patients with pulmonary fibrosis. In situ RNA hybridization with amplification confirms the co-existence of two distinct populations of alveolar macrophages in the same anatomical niche in patients with pulmonary fibrosis. The increased heterogeneity within alveolar macrophages in fibrotic lungs is potentially clinically relevant [33]. Related to this, our present study used silica inhalation rat models, and typical pathological changes in silicosis were summarized as follows: the occurrence of macrophage alveolitis, cellular nodules, and fibrotic cellular nodules. The biphasic pulmonary responses in rats exposed to silica exhibited continuous progressive fibrotic responses, including an increase in minute cellular nodules in the early stage (Week 2) and the formation of fibrotic cellular nodules in the later stage (Week 24) and the pathological changes mainly fasten on macrophages and AT2.cells. Numerous studies, including that conducted by our group, have used the presence of fibrotic nodules as a criterion to evaluate the severity of pulmonary fibrosis [4,5,12,14,15,20,21,24,34,35]. We attempted to explore the mechanisms of silicosis by using myofibroblast differentiation [24] and EMT models [14,21] in vitro or applying proteomics and genomics [4,5] in silicotic rats with long-term exposure to silica. Images of the present study showed that, as the silica exposure time was extended, macrophages, AT2 cells, and pulmonary vascular endothelial cells lost their phenotype, and AT2 cells around nodules were arranged in a cable. Notably, α-SMA was positively expressed in macrophages, and the co-expression of vimentin and ABCA3 in the nodules was difficult to find. That is, the occurrence of EMT in vivo was difficult to prove, and the result of our study was similar to that reported by Yamada [36].

The most important line of defense in the lower respiratory tract is made up of macrophages, macrophages phagocytic silica; then, silica and water form silicate, leading to the increased permeability of the lysosomal membrane and rupture. A lysosome burst release of lysosomal enzymes results in macrophages disintegrating autolysis, releasing silica dust simultaneously. A vicious cycle is then created. Some unleashed silica is transported away by lymphatic circulation. However, in this study, with the extension of silica-exposure time, the proportion of macrophages which contained silica in nodules gradually decreased. The presence of silica-free macrophages in and around nodules under polarized light microscopy. These silica-free macrophages need to be investigated to figure out the reason why the patients’ lung fibrosis continues to progress even if they have left the silica exposure environment. Silica-bearing macrophages present antigens to T lymphocytes, causing a series of subsequent inflammatory and immune responses. Macrophages have often been considered to possess activation properties and functions specific for the stimulus but independent of their location; thus, macrophage functions in homeostasis and disease are transposed from one tissue to another [37]; this may explain the presence of silica-free macrophages around the nodules.

Pulmonary transplantation of human-induced pluripotent stem cell-derived macrophages ameliorates pulmonary alveolar proteinosis [38]. Fibrogenesis-related research indicates that anti-inflammatory therapy alone only slightly affects the progression of fibrogenesis even if the inflammation is well-controlled [39], suggesting that the progression of silicosis fibrosis is not only caused by inflammation. Previous research has shown that, unlike acute silicosis, chronic exposures to occupationally relevant doses of silica cause a significant reduction in lung inflammation and an increase in the expression of anti-apoptotic, rather than proapoptotic markers in the lung, may result from the interaction between nuclear factor kappa-B-p50 and Bcl-3. Unlike acute silicosis, chronic silicosis is associated with limited inflammation and an anti-apoptotic response. This finding may explain the resistance of human silicosis to anti-inflammatory therapies [40]. However, the formation of fibrotic nodules indicates the irreversibility and progression of pulmonary fibrosis. Thus, ignoring early lesions (minute cellular nodules) is the main obstacle to the diagnosis and treatment of silicosis. Meanwhile, many antifibrotic drugs exhibit no significant efficacy in clinical practice. Screening of early markers of silicosis has recently been studied, and progress to a certain extent has been accomplished [41,42]. However, early morphological data on silicosis, which are vital for the diagnosis and treatment of the disease, remain scarce. These early lesions should be paid attention to, and research focus should be directed toward them.

## Data Availability

Not applicable.

## Supplementary Materials

The following supporting information can be downloaded at: https://www.mdpi.com/article/10.3390/vetsci9060251/s1, Figure S1: HE staining for silicotic nodules in rats exposed to silica for 2, 4, 8, 12, 16, and 24 weeks; Figure S2: positive expression of vimentin in rats with exposure to silica via inhalation for 12 weeks; Figure S3: VG staining and PAS staining in rats exposed to silica via inhalation for 4, 12, and 24 weeks; Figure S4: co-expression of vimentin and ABCA3 in silicotic nodules; Figure S5: positive expression of S100A4 in rats exposed to silica via inhalation for 2, 4, 8, 12, 16, and 24 weeks.
