# Supplementary material for: Minute Cellular Nodules as Early Lesions in Rats with Silica Exposure via Inhalation
Source: Vet Sci. 2022 May 25;9(6):251. doi: 10.3390/vetsci9060251 (PMC9227695; doi:10.3390/vetsci9060251)
Supplement: Supplementary file 1 [file vetsci-09-00251-s001.zip › vetsci-1721971-supplementary.pdf]

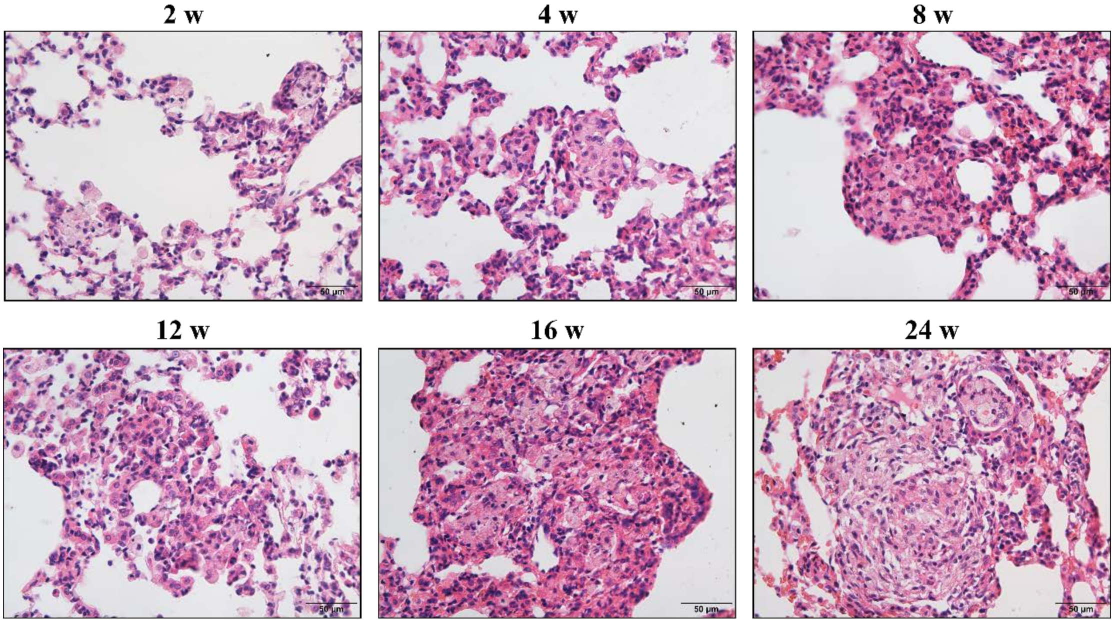

Figure S1. HE staining for silicotic nodules in rats exposed to silica for 2, 4, 8, 12, 16, and 24 weeks. Rats exposed to silica at the 2–16 weeks time points show "cellular nodules+ macrophage alveolitis"; rats exposed to silica via inhalation for 24 w show "fibrotic cellular+ cellular nodules". (Bar=50 μm)

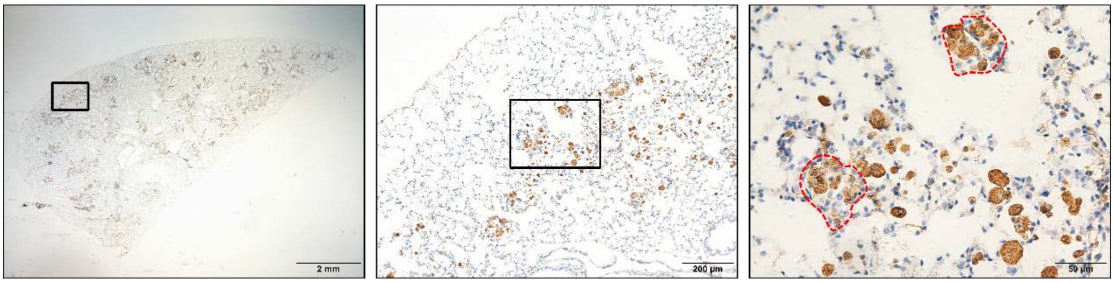

Figure S2. Positive expression of vimentin in rats with exposure to silica via inhalation for 12 weeks. Macrophage alveolitis consists of multiple free macrophages in the alveolar cavity or septum (in the black box). Cellular nodules consist of closely adjacent macrophages encircled by a red dotted line. (Bar=2 mm, 200 μm, and 50 μm)

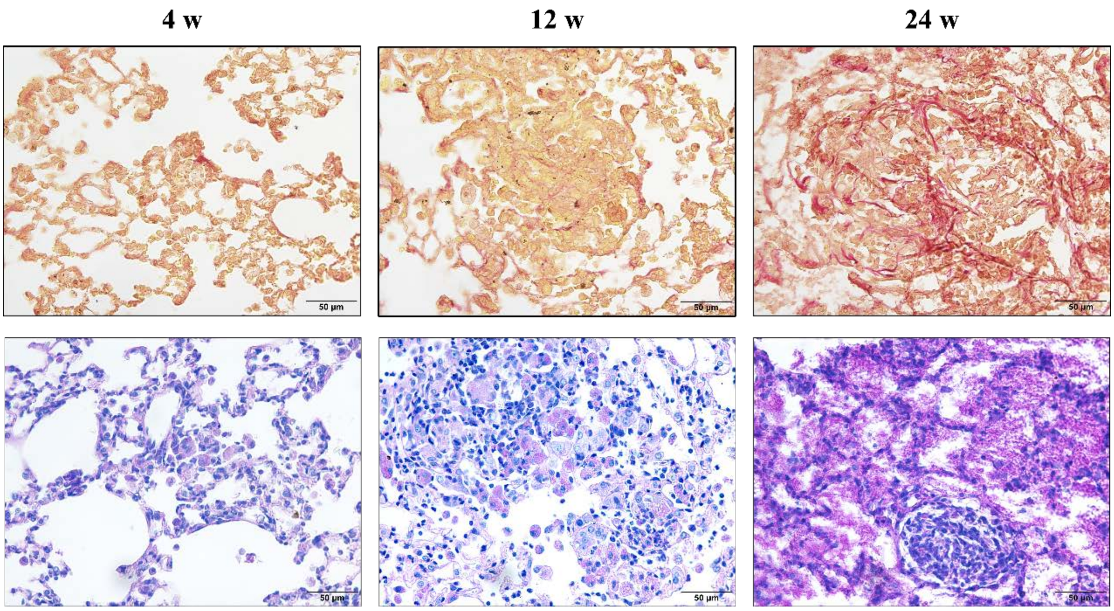

Figure S3. VG staining and PAS staining in rats exposed to silica via inhalation for 4, 12, and 24 weeks. With the extension of silica exposure time, collagen deposition and lipoproteinosis became increasingly prominent. (Bar=50 μm)

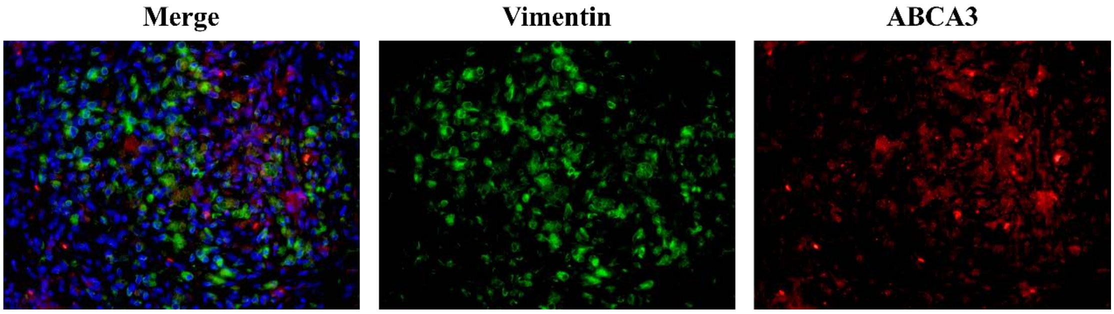

Figure S4. Co-expression of vimentin and ABCA3 in silicotic nodules. Both AT2 cells (red) and macrophages (green) are present in silicotic nodules, but cells co-expressing vimentin and ABCA3 are hardly visible. (Bar=50 μm)

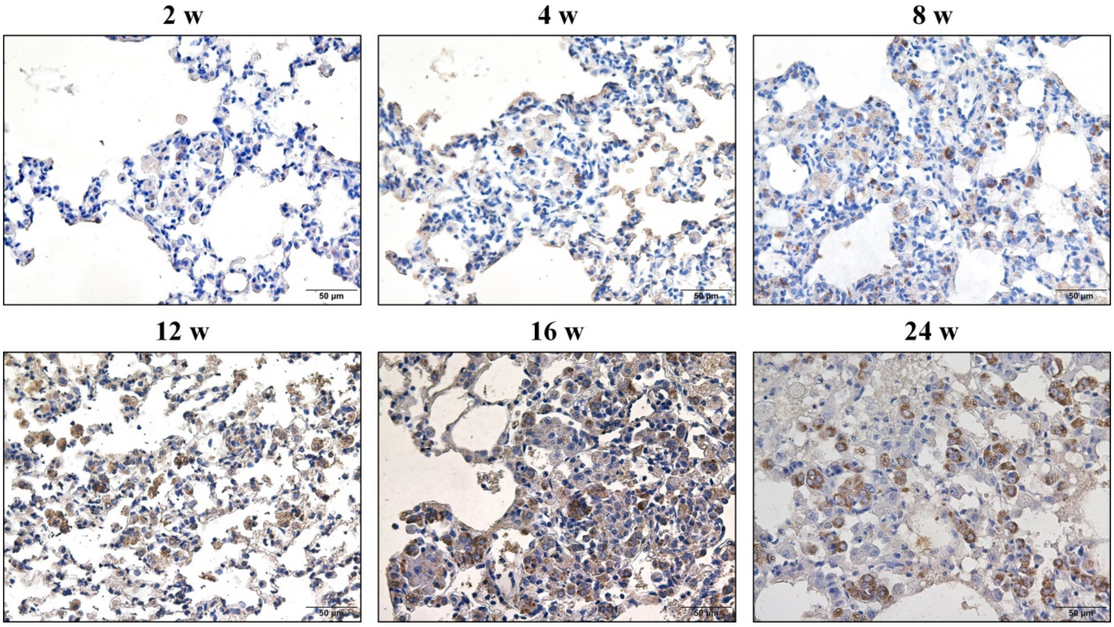

Figure S5. Positive expression of S100A4 in rats exposed to silica via inhalation for 2, 4, 8, 12, 16, and 24 weeks. S100A4 is positively expressed in macrophages in rats exposed to silica from the 2–24 weeks time points. (Bar=50 μm)
